# Inhibition of Lung Cancer Proliferation by Wogonin is Associated with Activation of Apoptosis and Generation of Reactive Oxygen Species

**DOI:** 10.4274/balkanmedj.galenos.2019.2019.7.75

**Published:** 2019-12-20

**Authors:** Chengyang Wang, Chuangcheng Cui

**Affiliations:** 1 Department of Radiology, The Ninth Hospital of Xi’an Affiliated Hospital of Xi’an Jiaotong University, Xi’an, Shaanxi, China

**Keywords:** Apoptosis, caspases, cell study, cytotoxicity, lung cancer, wagonin

## Abstract

**Background:**

Lung cancer has a very high incidence rate and is one of the commonly diagnosed tumors in developed countries.

**Aims:**

To investigate the effect of wogonin on A549 and A427 lung cancer cells and explore the mechanism involved.

**Study Design:**

Cell study.

**Methods:**

The cytotoxicity effect of wogonin on A549 and A427 lung cancer and BEAS-2B cells was assessed by MTT assay. The onset of apoptosis was assessed by flow cytometry using Annexin V FITC/PI staining. Western blotting was used for the determination of changes in apoptotic protein expression.

**Results:**

Wogonin treatment exhibited cytotoxicity effect selectively on A549 and A427 cells without affecting BEAS-2B normal lung cells. The viability of A549 and A427 cells was reduced to 31% and 34%, respectively, on treatment with 50 μM of wogonin; however, there was no significant reduction in BEAS-2B cell viability on treatment with the same concentration of it. Moreover, the percentage of apoptotic A427 cells showed a significant (p<0.049) increase on treatment with wogonin. Furthermore, the treatment led to a marked increase in the activation of caspase 3/8/9 and the generation of reactive oxygen species (ROS) at 72 h in A427 cells. Digital tomosynthesis studies showed a marked reduction in tumor development on treatment with wogonin.

**Conclusion:**

Wogonin treatment specifically exhibits a cytotoxic effect on lung cancer cells and this effect is associated with activation of apoptosis and generation of reactive oxygen species.

Lung cancer has a very high incidence rate and is one of the commonly diagnosed tumors in developed countries (1). In the USA alone, more than 15 million patients with lung cancer were detected (1). Its treatment includes a primary tumor radical resection, followed by an adjuvant chemotherapy (2). Despite the development of modern techniques, lung cancer has very poor prognosis, and, in a majority of patients, tumor recurrence has been observed (2,3,4). The poor response of patients to currently available drugs demands discovery of novel and effective chemotherapeutic agents for lung cancer treatment. Reactive oxygen species (ROS) formed from various cellular metabolites play a vital role in suppression of cancer growth (5). Disturbance of equilibrium between anti-oxidants and ROS in the cells leads to oxidative stress (6). The ROS overgeneration acts as a signaling pathway for carcinoma cell apoptosis through DNA damage (5). It has been recognized that ROS serves as an anti-tumor molecule (6).

Plants play a vital role in drug discovery, since many of the secondary metabolites have shown pharmacological activities (7,8,9). *Scutellaria* belongs to *Labiatae* family consisting around 400 species of annual and perennial herbs (7). The *Scutellaria* extract has been used for the treatment of inflammation, allergy, and hepatitis in traditional system of medicine (8). Phytochemical investigation led to the isolation of flavonoid and terpenoid compounds from this plant, with the major compounds identified being baicalin, baicalein, and wogonin (9). In consistence with the reported activity of flavonoid compounds, these molecules showed radical-quenching potential, anti-oxidant activity, and anti-tumor property (10). Moreover, the wogonin molecules have a flavonoid structure, which may bestow an anti-proliferative activity to it. Wogonin has been found to inhibit inflammatory activity of microglial cells by decreasing the generation of nitric oxide and cytokines (11). The present study was devised to investigate the effect of wogonin on lung cancer cell growth and proliferation. The study showed that wogonin is specific in inhibiting lung cancer cell proliferation without any toxicity against the normal pulmonary cells.

## MATERIALS AND METHODS

### Cell culture

The A549 and A427 lung cancer cells and BEAS-2B normal cells were obtained from the Chinese Academy of Sciences, Shanghai, China. The cell lines were maintained for 24 h in RPMI 1640 medium. The medium was supplemented with 10% fetal bovine serum and antibiotics (100 U/mL penicillin/streptomycin). The cell culture was performed under humid atmosphere of 95% air and 5% CO_2_ at 37 ˚C.

### Cell viability assay

The changes in A549, A427, and BEAS-2B cell proliferation on exposure to 5, 10, 15, 20, 25, 30, and 50 µM of wogonin were assessed using MTT assay. The cells at 2x104 cells/well density were distributed in 96 well plates and cultured for 24 h. Then, fresh medium mixed with 5, 10, 15, 20, 25, 30, and 50 µM of wogonin was added to the wells, and the plates were incubated for 72 h. After 72 h, 20 μL of MTT (0.5 mg/mL) solution was added to the wells and incubation was performed for further 4 h. The medium was then removed and 120 μL of dimethyl sulfoxide was added to each well. A microplate reader was used for the measurement of absorbance of each plate at 490 nm.

### EdU proliferation assay

The A549, A427, and BEAS-2B cells at 2x104 cells/well density were distributed in 96 well plates. The cells were treated with 5, 10, 15, 20, 25, 30, and 50 µM of wogonin for 72 h. The changes in cell proliferation were determined by EdU proliferation assay kit (Guangzhou RiboBio Co., Ltd., Guangzhou, China). Fluorescence microscope (Olympus IX51; Olympus Corporation, Tokyo, Japan) was used for observing the EdU stained cells.

### Analysis of cell morphology using Hoechst 33342 staining

The A427 cells at 2 x10^5^ cells/well concentration were distributed in 12 well plates and cultured for 24 h. The cells were incubated with 25, 30, and 50 µM of wogonin for 72 h, followed by washing twice with phosphate buffered saline (PBS). The cells were fixed with 4% paraformaldehyde for 20 min at room temperature, washed with PBS thrice, and subsequently stained for 20 min with Hoechst 33342 (10 μg/mL). Fluorescence microscope (Olympus Corporation) was used for detection of morphological changes in the cells.

### Detection of apoptosis using Annexin V FITC/PI staining

Apoptosis in A427 cells on treatment with wogonin was detected by flow cytometry using Annexin V FITC/PI kit. The cells at 72 h of treatment with 25, 30, and 50 µM of wogonin were harvested and washed using ice-cold PBS. The cells at 2 x10^5^ cells/mL density were suspended in 1X binding buffer. The cells were stained for 20 min with Annexin V FITC and PI solutions at room temperature under complete darkness. Flow cytometry (Beckman Coulter, Inc., Brea, CA, USA) was used for the analysis of the stained cells for apoptosis induction.

### Detection of ROS generation

For the detection of ROS generation in A427 cells, 2',7'-dichlorofluorescein-diacetate (DCFH-DA) was used. The cells were treated with 25, 30, and 50 µM of wogonin for 72 h, followed by washing with cold PBS thrice. The cells were then re-suspended in serum-free culture medium mixed with 10 μM DCFH-DA. The stained cells were detected for ROS generation by flow cytometry.

### Western blot analysis

The cells after 72 h of treatment with 25, 30, and 50 µM of wogonin were lysed using radioimmunoprecipitation assay lysis buffer. The lysate was centrifuged at (12,000xg) for 15 min at 4 ˚C to collect the supernatants. The protein concentration in the cell lysates was analyzed using a Bicinchoninic acid protein assay kit (Thermo Fisher Scientific, Inc.). The 30 μg protein samples were resolved on sodium dodecyl sulfate-polyacrylamide gel electrophoresis (8%-12%) and subsequently transferred onto polyvinylidene difluoride membranes. The nonspecific sites in the membranes were blocked on incubation with 5% nonfat milk. The membranes were incubated overnight at 4 ˚C with primary antibodies: anti-LC3, anti-RIP3, anti-caspase 3, anti-LC3I, anti-LC3II, anti-caspase 8, ant-caspase 9, and anti-Poly (ADP-ribose) polymerase (PARP). After twice washing with PBS, the membranes were subjected to incubation with horseradish peroxidase conjugated goat anti rabbit secondary antibody at room temperature for 2 h. The enhanced chemiluminescence detection system (Pierce; Thermo Fisher Scientific, Inc.) was used for visualization of the protein bands. Ethical approval was obtained from the local ethics committee, and written informed consent was obtained from all patients (approval number: 201917).

### Statistical analysis

Data of the triplicate experiments were analyzed statistically using SPSS 17.0 software (IBM Corp., Armonk, NY, USA) and were presented as mean ± standard deviation. The data comparison was performed using ANOVA, followed by Tukey's or Dunnett's test. A p<0.05 was considered as statistically significant.

## RESULTS

### Wogonin inhibits A549 and A427 lung cancer cell viability without affecting BEAS-2B normal cells

**Figure 1:**
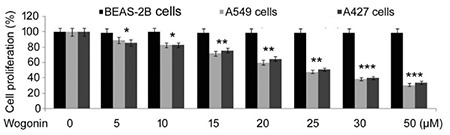
Effect of wogonin on lung cell viability. A549, A427 cancer cells, and BEAS-2B normal cells were exposed to different doses of wogonin. The changes in cell viability by wogonin were assessed by MTT assay. *p<0.048, **p<0.019, and ***p<0.011 vs control cells.

The proliferation of BEAS-2B normal lung cells was not affected on exposure to 5, 10, 15, 20, 25, 30, and 50 µM of wogonin (Figure 1). However, A549 and A427 cell viability showed a significant (p<0.048) decrease on treatment with wogonin for 72 h in a dose-dependent manner. On treatment with 5, 10, 15, 20, 25, 30, and 50 µM of wogonin, the viability of A549 cells reduced to 89%, 83%, 72%, 60%, 48%, 39%, and 31%, respectively, and that of A427 cells decreased to 86%, 83%, 76%, 65%, 51%, 40%, and 34%, respectively.

### Wogonin reduces A427 cell count

**Figure 2:**
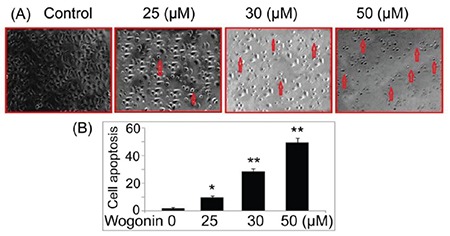
Effect of wogonin on lung cancer cell morphology. (A) The A427 cells were exposed to 25, 30, and 50 μM doses of wogonin. The cell morphological changes by wogonin were examined by microscopy. Images were taken at magnification of x200. The arrows indicate apoptotic cells. (B) Quantification of cell apoptosis. *p<0.05 and **p<0.02 vs control cells.

The micro¬scopic observation showed rounding of A427 cells on treatment with wogonin (Figure 2). The A427 cell count decreased significantly on treatment with 25, 30, and 50 µM of wogonin at 72 h.

### Wogonin induces apoptosis of A427 cells

**Figure 3:**
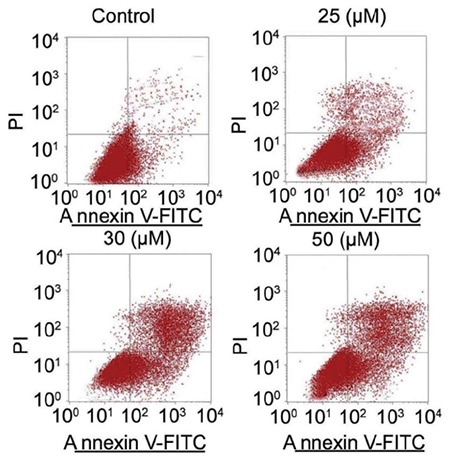
Wogonin treatment of lung cancer cells causes apoptosis induction. The A427 cells exposed to 25, 30, and 50 μM of wogonin were examined by flow cytometry after Annexin V FITC/PI staining. Images were taken at magnification of x200.

Annexin V FITC/PI staining of control A427 cells showed normal nuclear shape and weak blue fluorescence (Figure 3). However, the wogonin treated A549 and A427 cells showed bright blue fluorescent granules and chromosomal condensation. The extent of bright blue fluorescence increased markedly in A427 cells on increasing the dose of wogonin from 25 to 50 µM.

### Effect of wogonin on the expression of apoptotic proteins in A427 cells

**Figure 4:**
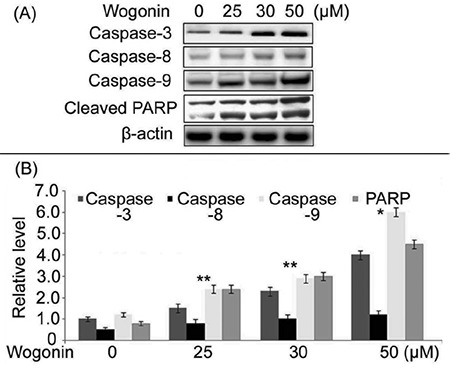
Expression of apoptotic proteins in A427 cells on treatment with wogonin. The cells were treated with 25, 30, and 50 μM of wogonin for 72 h. The protein concentration was determined by western blot assay using β actin as internal control.

The expression of procaspases 8/9/3 and cleaved PARP in A549 and A427 cells on treatment with wogonin was assessed by western blotting (Figure 4). A significant increase in caspases 8/9/3 expression was seen in A427 cells on treatment with 25, 30, and 50 µM of wogonin. Likewise, the expression of cleaved PARP was also found to be increased.

### Wogonin induces ROS generation in A427 cells

**Figure 5:**
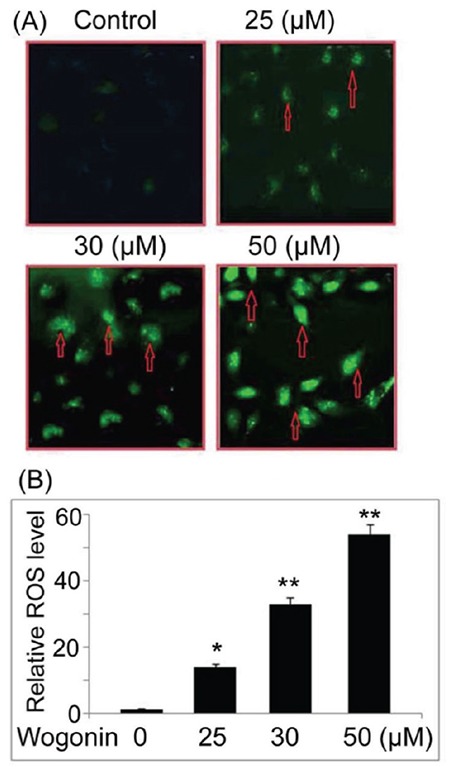
Effect of wogonin on reactive oxygen species (ROS) generation in lung cancer cells. (A) The cells exposed to different doses of wogonin were labeled with DCFH-DA and then examined by flow cytometry for ROS generation. The arrows indicate ROS generation. (B) Quantification of the ROS level. *p<0.05 and **p<0.02 vs control cells. DCFH-DA: dichlorofluorescein-diacetate

The ROS generation in A427 cells on treatment with wogonin was analyzed using DCFH-DA probe by flow cytometry (Figure 5), and it was found to be promoted significantly by wogonin at 25, 30, and 50 µM in A427 cells at 72 h. Thus, wogonin promoted generation of ROS in lung cancer cells in a dose-dependent manner.

### Wogonin induces autophagy in A427 cells

**Figure 6:**
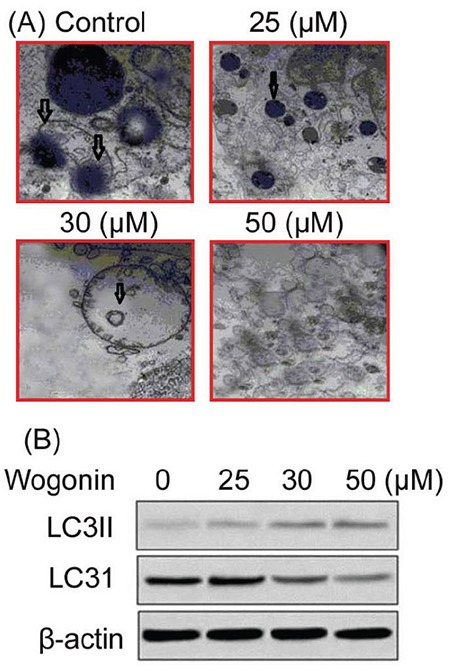
Effect of wogonin on autophagy induction in A427 cells. (A) The cells treated with different doses of wogonin were examined for acidic autophagic vacuole formation by fluorescence microscopy. Magnification, x200. The arrows indicate apoptotic autophagic vacuoles. (B) The levels of LC3I and LC3II in A427 cells treated with different doses of wogonin were analyzed by western blotting.

The acidic autophagic vacuole formation in A427 cells on treatment with wogonin was detected by fluorescence microscopy (Figure 6A). The formation of acidic autophagic vacuoles was found to increase significantly in A427 cells on treatment with 25, 30, and 50 µM doses of wogonin for 72 h. Moreover, the treatment with wogonin enhanced the expression of LC3II in these cells in a dose-dependent manner (Figure 6B). However, the levels of LC3I were decreased markedly in these cells on treatment with 25, 30, and 50 µM doses of wogonin.

## DISCUSSION

The present study demonstrated that wogonin exhibited an inhibitory effect on A549 and A427 carcinoma cell proliferation in a dose-dependent manner. Moreover, it did not exhibit any toxicity on BEAS-2B normal lung cells. Therefore, the present study suggests that wogonin specifically exhibits toxicity against lung cancer cells without affecting the normal epithelial cells.

Apoptosis, a complex process associated with the elimination of unwanted cells from the body, is controlled by several genes (12). The signaling pathway involved in apoptosis induction is linked to the activation of pro-caspases (13). In the present study, wogonin treatment reduced the proliferation of A549 and A427 carcinoma cells markedly in comparison to the control. The study investigated the mechanism of inhibition of lung cancer cell proliferation on treatment with wogonin. Flow cytometry showed that wogonin treatment markedly promoted the onset of apoptosis in A549 and A427 cells. Therefore, wogonin suppressed lung cancer cell proliferation by the activation of apoptotic signaling pathway. It has been well established that caspases play a vital role in arresting carcinoma growth by inducing cell apoptosis (14). The members of caspase family like caspase 2, -8, 9, and 10 (initiators) are involved in the activation of apoptotic cascade; while, caspase 3, 6, and 7 (executers) execute the process of apoptosis (15). In the present study, wogonin treatment enhanced the expression of both initiator as well as executer caspases in A549 and A427 cells. The expression of initiator caspase -8 and executer caspase -3 in A549 and A427 cells was markedly higher on treatment with wogonin. These findings proved that wogonin caused an induction of apoptosis in A549 and A427 cells through caspase-dependent pathway. The level of cleaved PARP in A549 and A427 cells was also promoted on treatment with wogonin. Moreover, an increased generation of ROS also acts as a signaling pathway for the activation of cell apoptosis (16). The higher concentration of ROS leads to DNA damage, oxidative stress, followed by cell apoptosis (17). Studies have shown that an upregulation of ROS generation in cells suppresses cell proliferation by inducing apoptosis (18). In the present study, the levels of ROS were markedly promoted in A549 and A427 cells with wogonin. Furthermore, an overgeneration of ROS in the cancer cells has been found to be linked with autophagy (19,20). In the present study, wogonin promoted acidic autophagic vacuole formation in A549 and A427 cells. There was a marked upregulation of LC3II expression in A549 and A427 cells on treatment with wogonin.

In summary, the present study demonstrated an anti-cancer potential of wogonin against lung cancer cells without any toxic effect on normal cells. The toxic effect of wogonin involved apoptosis induction, activation of caspases, and increased generation of ROS in A549 and A427 cells. Therefore, wogonin can be used to develop an effective treatment strategy in lung cancer.
